# Editorial: Neurological Soft Signs in Neuropsychiatric Conditions

**DOI:** 10.3389/fpsyt.2018.00736

**Published:** 2019-01-14

**Authors:** Pablo Toro, Johannes Schröder

**Affiliations:** ^1^Department of Psychiatry, School of Medicine, Pontificia Universidad Católica de Chile, Santiago, Chile; ^2^Advanced Center for Chronic Disease, School of Medicine, Pontificia Universidad Católica de Chile, Santiago, Chile; ^3^Section of Geriatric Psychiatry, Heidelberg University, Heidelberg, Germany

**Keywords:** neurological soft signs (NSS), neuropsychaitric disorders, schizophrenia, organic brain disorder, clinical tool

Neurological soft signs (NSS) are minor motor and sensory deficits that can be easily examined in any clinical examination (1). While the majority of studies investigated NSS in patients with schizophrenia, signs can be also demonstrated in affective and personality disorders and in patients with organic brain changes, such as HIV associated neurocognitive disorder (2) or Alzheimer's disease (3). Hence, NSS are not specific for any severe neuropsychiatric condition, but rather correspond to psychopathological symptoms, cognitive deficits and their underlying structural and functional brain changes.

Longitudinal studies in schizophrenia (4) found NSS to vary in the clinical course with severity of psychopathological symptoms in particular formal thought disorders, negative symptoms, or apathy. However, even in patients with a remitting course of the disorder, NSS do not normalize completely. Instead, NSS scores stay in between the low values characteristic for healthy controls and elevated values typical for patients with chronic schizophrenia, i.e., in the range that also applies to subjects with an increased genetic vulnerability toward the disorder. Among other findings, the decrease of NSS with remission of psychopathological symptoms under neuroleptic treatment demonstrate that NSS are not the merely a consequence of neuroleptic side effects. Instead, both NSS and an increased risk of neuroleptic side effects may be associated with chronicity of the disease (5).

Neuroimaging studies (6, 7) identified the sensorimotor cortices, the supplementary motor area, the basal ganglia, the thalamus and the cerebellum as important sites for NSS.

While the respective studies—among them probably the first fMRI study in schizophrenia (6) and a single longitudinal trail (8)—solely included patients with schizophrenia, results suggest that NSS refer to disseminated changes in the whole motor system in particular a cerebello-thalamo-prefrontal network (9) rather than to deficits in discrete cerebral sites. Similarly, NSS are associated with a wealth of cognitive deficits ranging from attention/psychomotor speed and executive dysfunctions to complex neuropsychological abilities, such as logical memory, autobiographic episodic memory, and theory of mind (10).

In the light of these findings, NSS in schizophrenia indicate a rather generalized deficit that refers to both, trait (i.e., genetic liability) and state (i.e., acuity of the disease process) related factors. Hence, NSS are a useful clinical tool to assess these factors and thus may help to identify subjects with an increased liability toward schizophrenia or patients with an increased risk for a new acute exacerbation of the disease.

These aspects of NSS were discussed and enhanced in the six contributions to the research topic. Ojagbemi et al. investigated extrapyramidal side effects in a large group of first- episode patients before and 3 months after initiation of treatment with flupenthixol decanoate, i.e., a conventional neuroleptic. Their results confirm and enlarge the previous observation that extrapyramidal signs occur even in patients who have never been medicated and support the hypothesis that NSS, poor treatment response, and motor side effects represent different aspects of the chronicity of the disorder.

Schäppi et al. from Bern investigated the effects of genetic liability on four aspects of motor dysfunction, namely NSS, abnormal involuntary movements, Parkinsonism, and fine motor function. When contrasted with a healthy control group, patients showed deficits in all four domains, while deficits in first-degree relatives were restricted to NSS including finger- tapping. This dissociation was assigned to a stronger genetic impact on NSS rather than on abnormal involuntary movements and Parkinsonism.

A significant increase of NSS with age was demonstrated by Herold et al. from the Heidelberg group in patients with chronic schizophrenia and alternatively referred to ongoing cerebral changes or to a greater susceptibility of patients in physiological aging. This remembrance to Kraepelinian concept of dementia praecox was mediated among other factors by a lower cognitive reserve in patients than healthy controls.

In their discussion of 29 longitudinal studies on NSS in schizophrenia published between 1966 and 2017, Bachmann and Schröder found NSS to share both, trait and state related aspects. Chronicity was the most important predictor for the longitudinal course of NSS, since all studies on chronic schizophrenia showed that NSS remained stable or deteriorated, whereas studies on patients with a remitting course found that NSS decreased over time.

Galindo et al. conceptualized NSS and DMN hyperconnectivity as neurobiological markers of schizophrenia. Both abnormalities were not only clearly confirmed in patients with schizophrenia but also—albeit to a lesser extend—in their unaffected relatives. Moreover, the study identified the caudate nucleus was identified as the potential gateway to the motor consequences of abnormal DMN connectivity.

By using the Finger Force Manipulandum (FFM), i.e., a sophisticated instrument to quantify key components of finger movements, Térémetz et al. analyzed multiple aspects of sensorimotor control—namely visuomotor precision, motor sequence recall and temporal regularity, and independence of finger movements—with respect to impaired manual dexterity in schizophrenia. Visuomotor precision and degree of individuation correlated with NSS scores, good diagnostic accuracy and responsiveness to treatment suggest that manual dexterity may represent a useful clinical marker in schizophrenia.

Findings from these studies have the potential to enhance our understanding of NSS in schizophrenia and other neuropsychiatric conditions, and may facilitate the development of new ways to establish an early prognosis. While NSS already can be used in the clinical work up of patients with schizophrenia, clinical studies in affective disorders and organic brain disorders still are scarce. Longitudinal studies are necessary to confirm that the characteristics of NSS established in schizophrenia can be transferred to affective disorders and organic psychosis and to establish the associations of the various aspects of NSS in the clinical course of neuropsychiatric conditions.

## Author Contributions

All authors listed have made a substantial, direct and intellectual contribution to the work, and approved it for publication.

### Conflict of Interest Statement

The authors declare that the research was conducted in the absence of any commercial or financial relationships that could be construed as a potential conflict of interest.
